# Baseline gut microbiota and short-term response to acupressure combined with thermal stimulation in maintenance hemodialysis patients with functional constipation: an exploratory responder analysis

**DOI:** 10.3389/fcimb.2026.1803544

**Published:** 2026-10-13

**Authors:** Saiping Chen, Mengqi Wu, Min Huang, Bin Lu, Jiarui Wang, Hong Liu, Yuqing Wang, Junyi Liu, Aiping Zhang, Yanqin Zhu, Fenggui Zhu, Xueyan Zeng, Shilei Chen, Xin Zhou, Riyang Lin

**Affiliations:** 1 Department of Nephrology, Hangzhou Traditional Chinese Medicine Hospital of Zhejiang Chinese Medical University, Hangzhou, Zhejiang, China; 2 Department of Respiratory and Critical Care Medicine, Lishui Hospital of Traditional Chinese Medicine Affiliated to Zhejiang University of Traditional Chinese Medicine, Lishui, China; 3 Xingqiao Street Community Health Service Center, Hangzhou, Zhejiang, China; 4 Department of Nephrology, Sichuan Second Hospital of Traditional Chinese Medicine, Chengdu, Sichuan, China; 5 Department of General Medicine, Hangzhou Xihu District Zhuantang Street Community Health Service Centre, Hangzhou, Zhejiang, China; 6 Tianshui Wulin Street Community Health Care Centre, Hangzhou, Zhejiang, China; 7 Key Laboratory of Kidney Disease Prevention and Control Technology, Hangzhou, Zhejiang, China

**Keywords:** 16S rRNA gene sequencing, acupressure, functional constipation, gut microbiota, maintenance hemodialysis, response heterogeneity, thermal stimulation

## Abstract

**Background:**

Constipation is common in patients undergoing maintenance hemodialysis (MHD), and individual responses to non-pharmacological interventions may vary substantially. This exploratory study investigated whether baseline gut microbiota characteristics were associated with short-term response to acupressure combined with thermal stimulation in MHD patients with functional constipation.

**Methods:**

This responder analysis was based on a randomized two-sequence crossover study. Clinical data and fecal samples obtained immediately before each participant entered the 14-day intervention period were analyzed. Participants with an increase of ≥1 bowel movement (BM) per week were classified as responders (R), and those with <1 BM/week as non-responders (NR). Baseline gut microbiota were characterized using 16S rRNA gene sequencing. Microbial diversity, taxonomic composition, and genus-level differential abundance were evaluated. ANCOM-BC2 was performed with adjustment for baseline BM/week, sex, and diabetes mellitus, followed by Benjamini–Hochberg false-discovery-rate correction and pseudocount sensitivity analysis.

**Results:**

Twenty-six participants were included, of whom 9 (34.6%) met the predefined response criterion. Across all participants, weekly BM frequency increased from 1.25 (1.00, 1.50) to 2.00 (1.00, 3.00) (P = 0.0003), while PAC-SYM total and stool-symptom subscale scores decreased (P = 0.0011 and P = 0.0013, respectively). Alpha-diversity did not differ significantly between R and NR groups, and beta-diversity showed no overall community separation (ANOSIM R=−0.044, P = 0.6751). *Lactonifactor* was nominally higher in responders in the unadjusted analysis (P = 0.015) but was not significant after multiple-testing correction (FDR = 0.86). In adjusted ANCOM-BC2 analysis, *Veillonella* (β=5.319, FDR = 0.0347) and *Lactobacillus* (β=3.621, FDR = 0.0459) were associated with responder status; however, neither association was robust to pseudocount sensitivity analysis. PICRUSt2 identified no significant between-group differences in predicted functional pathways.

**Conclusion:**

Baseline microbial diversity and community structure were similar between responders and non-responders. *Veillonella* and *Lactobacillus* showed adjusted associations with response, but neither was robust in sensitivity analysis. These findings should therefore be regarded as exploratory rather than predictive. Larger longitudinal studies are needed to validate these findings and determine their clinical relevance.

## 1 Introduction

Maintenance hemodialysis (MHD) is a life-sustaining kidney replacement therapy for patients with end-stage kidney disease (ESKD). Constipation is highly prevalent in this population and is influenced by multiple factors, including dietary and fluid restrictions, reduced physical activity, the use of phosphate binders and potassium-binding agents, suppression of the urge to defecate during dialysis sessions, autonomic dysfunction, and alterations in the intestinal microenvironment (Yasuda et al., 2002; Zhang et al., 2022; Park et al., 2025). Reported prevalence rates of constipation in patients receiving hemodialysis range from approximately 25% to more than 60%. In addition to causing substantial discomfort, constipation is associated with impaired quality of life, sleep disturbance, depressive symptoms, and reduced treatment adherence in dialysis patients (Zhang et al., 2013; Bulbul et al., 2022; Zhu and Wang, 2025). More importantly, observational studies have suggested that lower defecation frequency and constipation may be associated with adverse cardiovascular and mortality outcomes, emphasizing the clinical importance of effective bowel management in this vulnerable population (Honkura et al., 2016; Park et al., 2025).

Accumulating evidence suggests that the gut microbiota may be relevant to both chronic kidney disease and constipation. Patients receiving maintenance dialysis exhibit substantial alterations in the intestinal microbial ecosystem, including changes in microbial composition and metabolic capacity, reductions in some short-chain fatty acid-producing taxa, and expansion of protein-fermenting or potentially pathogenic microorganisms (Simões-Silva et al., 2018; Wang et al., 2020; Li et al., 2025). These alterations may interact with impaired intestinal barrier function, chronic inflammation, and the accumulation of gut-derived uremic solutes. Constipation itself may further modify the intestinal ecological environment by prolonging colonic transit time and increasing the duration of contact between intestinal contents and the microbial community (Ramezani et al., 2016). Consistent with this concept, our previous cross-sectional study demonstrated differences in gut microbial characteristics between MHD patients with and without constipation (Zhang et al., 2024a). Thus, the intestinal microbiota may represent one component of the heterogeneous biological background underlying constipation in patients undergoing dialysis.

The long-term pharmacological management of constipation in MHD patients can be challenging because of polypharmacy, electrolyte disturbances, fluid restrictions, comorbidities, and concerns regarding the prolonged use of certain laxatives. Consequently, non-pharmacological approaches have attracted increasing interest. Acupoint-based interventions, including acupressure and related forms of acupuncture, have demonstrated potential benefits for gastrointestinal symptoms and constipation, while thermal stimulation is also commonly incorporated into traditional external therapies (Yang et al., 2022; Tan et al., 2025). Importantly, emerging evidence suggests that somatic stimulation at acupoints may have effects extending beyond local mechanical stimulation. Experimental studies of electroacupuncture have demonstrated changes in gut microbial composition and microbial metabolites. In a mouse model of functional constipation, electroacupuncture improved intestinal transit, altered the intestinal microbial community, and increased butyrate production; the attenuation of its effect in microbiota-depleted animals further suggested that the gut microbial environment may contribute to the physiological response to this form of acupoint stimulation (Xu et al., 2023). More recently, a preclinical study using modified meridian-guided acupoint pressing reported alterations in gut microbial diversity and community structure after manual acupoint stimulation, providing preliminary evidence that non-invasive acupoint pressing itself may also influence the intestinal microbial ecosystem, although these findings were obtained in an animal model and require clinical confirmation (Jiang et al., 2026).

Evidence is also emerging for an association between thermal acupoint therapies and the gut microbiota. Moxibustion is not equivalent to the controlled thermal stimulation used in the present study because it combines thermal effects with additional physical and chemical components; nevertheless, it provides relevant evidence that sustained peripheral thermal stimulation at selected body sites may be accompanied by changes in intestinal microbial composition. A systematic review and meta-analysis including three clinical and 20 preclinical studies found that acupuncture and/or moxibustion was associated with changes in specific microbial taxa, although effects on overall α-diversity were inconsistent and substantial heterogeneity was present across disease models and interventions (Bae et al., 2024). Of particular relevance to kidney disease, a randomized study in patients with chronic renal failure undergoing peritoneal dialysis reported that 12 weeks of moxibustion was accompanied by improvements in gastrointestinal function and alterations in gut microbiota structure (Yao et al., 2025). Clinical and experimental studies in other diseases have similarly reported microbiota changes following moxibustion, but the existing evidence remains heterogeneous and does not establish that isolated heat stimulation alone is responsible for these microbial effects.

Given the close association between gut microbiota, constipation, and the uremic intestinal environment, baseline microbial characteristics may represent one of the biological factors related to this response heterogeneity. However, whether pretreatment gut microbiota profiles differ between responders and non-responders to acupressure combined with thermal stimulation remains unclear. Therefore, this study characterized the baseline gut microbiota of MHD patients with functional constipation using 16S rRNA gene sequencing and compared microbial features between patients who did and did not achieve a predefined short-term clinical response after a 14-day intervention. The findings may provide preliminary evidence for understanding individual differences in treatment response and support future research on more individualized non-pharmacological management of constipation in the hemodialysis population.

## 2 Materials and methods

### 2.1 Study design and study participants

This study was an exploratory responder analysis based on a randomized two-sequence crossover study conducted in patients with functional constipation undergoing maintenance hemodialysis. Between December 2024 and June 2025, consecutive patients receiving MHD at the Hemodialysis Center of Hangzhou Hospital of Traditional Chinese Medicine were screened for eligibility. A total of 29 eligible participants were consecutively enrolled and subsequently randomized to an intervention-first group (IF group, n=15) or a control-first group (CF group, n=14). Participants in the IF group received acupressure combined with controlled thermal stimulation for 14 days followed by 14 days of routine hemodialysis care, whereas participants in the CF group first received 14 days of routine care followed by the same 14-day intervention. The total observation period was 28 days.

For the present analysis, clinical characteristics and fecal samples obtained immediately before each participant entered the 14-day intervention period were used as baseline data. Participants were subsequently classified as responders (R) or non-responders (NR) according to the predefined change in weekly bowel movement frequency during the intervention period. Of the 29 initially enrolled participants, two did not complete the prescribed acupressure intervention and one changed the laxative regimen during the study period. The remaining 26 participants were included in the final responder analysis, and their pre-intervention gut microbiota characteristics were compared between responders and non-responders.

The inclusion criteria were as follows:

1. Meeting the diagnostic criteria for stage 5 chronic kidney disease (CKD) as specified in the KDIGO 2024, i.e. estimated glomerular filtration rate (eGFR) < 15 mL·min^-1^·(1.73 m²)^-1^, and undergoing regular maintenance hemodialysis for at least 6 months.
2. Meeting the diagnostic criteria for functional constipation in the Rome IV Criteria, with symptom onset for at least 6 months and the presence of two or more typical constipation symptoms over the past 3 months.
3. Aged 18 to 75 years, regardless of gender.
4. Having a stable clinical condition, no acute complications, and the ability to cooperate with the intervention and follow-up assessments.
5. For patients taking laxatives, probiotics, prebiotics or other intestinal modulators: the aforementioned medications must have been used stably for at least 4 weeks prior to enrollment, with no changes in the brand, dosage or administration frequency during the study period.
6. No use of antibiotics, immunosuppressants or other drugs that significantly interfere with the intestinal flora within the past month.
7. Having provided written informed consent and voluntarily participating in the study.

The exclusion criteria were as follows:

1. Comorbid with severe organic diseases of the digestive system, including intestinal obstruction, intestinal neoplasms, inflammatory bowel disease (IBD), active peptic ulcers, or severe hemorrhoids.
2. Comorbid with severe failure of vital organs such as the heart, brain, liver and lungs, or suffering from systemic diseases including active infections or malignant tumors.
3. History of dosage adjustment or newly added use of laxatives, probiotics, prebiotics, or other intestinal modulators within 1 month prior to enrollment.
4. Presence of mental disorders, cognitive impairment, or consciousness disturbance, with an inability to accurately report discomfort or cooperate with the intervention and follow-up assessments.
5. Pregnant or lactating women.
6. Presence of skin damage, infection, ulceration, or severe pruritus at acupoint sites, rendering the patient unsuitable for thermal stimulation procedures.
7. A history of allergic reactions or significant intolerance to thermal stimulation or acupoint pressing procedures.
8. Withdrawal from the study during the research period, or incomplete collection of clinical data and biological samples.

### 2.2 Clinical data collection and potential confounders

Demographic and clinical data were obtained from study records and clinical assessments. The variables available for the present analysis included age, sex, body mass index (BMI), dialysis vintage, dialysis frequency, dialysis modality, dialysis adequacy assessed by Kt/V, primary kidney disease, hypertension, diabetes mellitus, serum calcium and phosphorus levels, laxative use, baseline weekly bowel movement frequency (BM/week), and constipation symptom severity assessed using the Patient Assessment of Constipation Symptoms (PAC-SYM), including the total score and stool-symptom subscale score. Particular attention was paid to factors that could potentially influence bowel habits or gut microbial composition. Laxative use was specifically recorded, and participants receiving laxatives before enrollment were required to maintain a stable regimen throughout the study without initiating a new laxative or changing the existing regimen.

### 2.3 Intervention method

#### 2.3.1 Acupoint selection

The selected acupoints included Daheng (SP15) and Chengshan (BL57). The localization of acupoints was performed in accordance with the national standard GB/T 12346–2021 Nomenclature and Location of Acupoints.

#### 2.3.2 Acupoint pressing

Prior to the initiation of the study, the researchers conducted standardized training for patients on acupoint localization, pressing intensity and operational precautions. Patients performed the acupoint pressing independently at home or after hemodialysis sessions, with the target acupoints being Daheng (SP15) and Chengshan (BL57). It was advisable for patients to experience a local sensation of soreness and distension, warmth and comfort when pressing each acupoint. Each acupoint was pressed for approximately 30 seconds, followed by a 5-second rest, repeated for 3 cycles; the procedure was performed twice a day for 14 consecutive days. During the study, the research team uniformly provided pressing sticks, and patients could choose to use the sticks or press directly with the finger pulp based on personal habits. The researchers conducted regular follow-up visits to document patients’ operational compliance and adverse reactions.

#### 2.3.3 Thermal stimulation

Prior to the initiation of the study, researchers explained the principles and precautions of thermal stimulation to patients and instructed them to master the correct usage method. Patients performed thermal stimulation at home, after hemodialysis sessions, or immediately following acupoint pressing, using temperature-controllable electric heating devices uniformly provided by the research team. The temperature was set in the range of approximately 40–45 °C, with each stimulation session lasting 20 minutes; the procedure was conducted twice a day for 14 consecutive days. During use, the local skin was required to be kept clean and dry, and the device was not to be applied to damaged, infected skin or the area around hemodialysis catheters. If a marked burning sensation, skin flushing or other discomforts occurred, the stimulation was to be stopped immediately. Patients could appropriately adjust the application duration according to their own tolerance, with the criterion of local warm comfort without burning pain. Researchers conducted regular follow-up visits to document the usage compliance and adverse reactions to the intervention. To balance clinical ethics and participant compliance, patients were permitted to continue their prior constipation-related treatments during the study period, and these treatments were required to be strictly standardized and documented in detail.

### 2.4 Outcome indicators

#### 2.4.1 Bowel movement frequency

Participants recorded bowel movements daily. Bowel movement frequency was expressed as the number of bowel movements per week (BM/week). Pre-intervention BM frequency was defined as the average weekly bowel movement frequency during the 2 consecutive weeks immediately preceding the intervention, whereas post-intervention BM frequency was defined as the weekly bowel movement frequency during the second week of the 14-day intervention. The change in bowel movement frequency was calculated as:


ΔBM/week = BM/week during the second intervention week−mean BM/week during the 2−week pre−intervention period.

For the prespecified responder analysis, participants with an increase of ≥1 bowel movement per week (ΔBM/week ≥1) were classified as responders (R), whereas those with ΔBM/week <1 were classified as non-responders (NR). An increase of at least one complete spontaneous bowel movement per week from baseline has been widely incorporated into responder definitions in clinical trials of chronic constipation and has also been used to characterize treatment response in acupuncture studies (Yang et al., 2017; Xiong et al., 2025). Because the present study recorded overall bowel movement frequency rather than complete spontaneous bowel movements, the ≥1 BM/week threshold was used as an operational criterion for exploratory response stratification rather than as a formally validated clinical responder endpoint.

#### 2.4.2 Assessment of constipation symptoms

Constipation-related symptoms were assessed using the Patient Assessment of Constipation Symptoms (PAC-SYM). The pre-intervention PAC-SYM score reflected participants’ overall symptoms during the 2 consecutive weeks immediately preceding the intervention, whereas the post-intervention score was obtained at the end of the second intervention week. The PAC-SYM total score and stool-symptom subscale score were recorded, and their changes from pre-intervention to post-intervention were evaluated as secondary clinical outcomes.

#### 2.4.3 Gut microbiota measures

Baseline gut microbiota characteristics were assessed using 16S rRNA gene sequencing of fecal samples collected immediately before the intervention. Microbiota outcomes included α-diversity indices (Shannon, Simpson, and Chao1), β-diversity based on community dissimilarity, and taxonomic composition and relative abundance, with the primary taxonomic analyses focused on the phylum and genus levels.

### 2.5 Fecal sample collection and detection

#### 2.5.1 Sample collection and preservation

Prior to the intervention, sterile sampling boxes with labeled information were distributed to the participants. Before defecation, participants placed sterile paper in the toilet bowl; after defecation, they collected 50–100 mg of fresh feces into the sampling boxes using sampling spoons, making every effort to avoid contamination of the samples by urine, toilet bowl surfaces and other substances during the collection process. After sealing, the samples were transported at low temperature with ice packs and transferred to a -80 °C refrigerator for cryopreservation within the shortest possible time (a maximum of 4 hours). Upon completion of all sample collections, the samples were sent to the High-Throughput Laboratory of Hangzhou Guangke Ande Technology Co., Ltd. for high-throughput sequencing analysis under cryopreserved conditions.

#### 2.5.2 Main reagents and instruments

Fecal DNA Extraction Kit (MJYH, Shanghai, China); NanoDrop 2000 Spectrophotometer (Thermo Fisher Scientific, USA); FastPfu Polymerase (TransGen Biotech, Beijing, China); T100 Thermal Cycler (Bio-Rad Laboratories, USA); Synergy HTX Microplate Reader (BioTek Instruments, Inc., USA); NEXTFLEX Rapid DNA-Seq Kit (Bioo Scientific, USA); JY600C Dual-stable Timed Electrophoresis Apparatus (Beijing Junyi Dongfang Electrophoresis Equipment Co., Ltd., Beijing, China).

#### 2.5.3 16S rRNA gene sequencing and analysis

Total genomic DNA of the microbial community in fecal samples was extracted according to the operating instructions of the Fecal DNA Extraction Kit. Subsequently, the integrity of the extracted genomic DNA was detected by 1% agarose gel electrophoresis, and the DNA concentration and purity were determined using a NanoDrop 2000 Spectrophotometer. Using the extracted DNA as the template, PCR amplification of the V3-V4 variable regions of the 16S rRNA gene was performed with the specific primers 338F (5’-ACTCCTACGGGAGGCAGCAG-3’) and 806R (5’-GGACTACHVGGGTWTCTAAT-3’) carrying Barcode sequences. After pooling the PCR products of the same sample, the target fragments were recovered by 2% agarose gel electrophoresis, and the recovered products were purified. The fragment sizes were detected via 2% agarose gel electrophoresis, and quantitative analysis of the purified products was conducted using a Synergy HTX Microplate Reader. The purified PCR products were used to construct libraries with the NEXTFLEX Rapid DNA-Seq Kit, and high-throughput sequencing was performed on the Illumina NextSeq 2000 PE300 platform.

Quality control of the paired-end raw sequencing reads was carried out using Fastp software, and sequence assembly was performed with FLASH software. Primer sequence identification and removal were then conducted using cutadapt software to obtain primer-free Clean Reads. Denoising was performed via the DADA2 method in QIIME2 software to generate Amplicon Sequence Variants (ASVs). After paired-end sequence merging and chimeric sequence removal, the final valid Non-chimeric Reads were obtained. Quality control of the paired-end raw sequencing reads was carried out using Fastp software, and sequence assembly was performed with FLASH software. Primer sequence identification and removal were then conducted using cutadapt software to obtain primer-free Clean Reads. Denoising was performed via the DADA2 method in QIIME2 software to generate Amplicon Sequence Variants (ASVs). After paired-end sequence merging and chimeric sequence removal, the final valid Non-chimeric Reads were obtained. Taxonomic annotation was implemented by aligning the sequences with the Silva 16S rRNA gene database (v138) using the RDP Classifier, with a 70% confidence threshold as the criterion; statistical analysis of the microbial community composition of each sample was subsequently performed at different taxonomic levels. Functional prediction analysis of the 16S rRNA gene sequences was conducted using PICRUSt2 software (version 2.2.0).

### 2.6 Statistical analysis

Continuous clinical variables are presented as median (Q1, Q3), and categorical variables as n (%). Between-group comparisons of continuous variables were performed using the Mann-Whitney U test, while categorical variables were compared using Fisher’s exact test because of the small sample size and sparse cells. Pre- and post-intervention clinical outcomes in the overall analysis population were compared using the Wilcoxon signed-rank test. All clinical tests were two-sided, with P <0.05 considered statistically significant.

α-diversity indices were calculated using Mothur, and between-group differences were assessed using the Wilcoxon rank-sum test. β-diversity was evaluated using principal coordinates analysis (PCoA) based on Bray-Curtis dissimilarity and non-metric multidimensional scaling (NMDS) based on Unweighted UniFrac distance. Overall community differences between R and NR groups were assessed using analysis of similarities (ANOSIM).

Genus-level differential abundance was analyzed using ANCOM-BC2 (version 2.8.1) with raw genus-level sequencing counts as input (Lin and Peddada, 2024). Genera with a prevalence >=20% and mean relative abundance >=0.01% were retained, leaving 81 genera for analysis. The primary model included response status as the variable of interest and adjusted for baseline BM/week, sex, and diabetes mellitus; the NR group, male sex, and absence of diabetes were used as reference categories. Benjamini-Hochberg false discovery rate (FDR) correction was applied across tested genera, with FDR <0.05 used as the primary multiple-testing threshold. ANCOM-BC2 pseudocount sensitivity analysis was additionally examined to assess robustness to zero handling.

Because the unadjusted descriptive abundance of *Lactobacillus* differed in direction from the fully adjusted ANCOM-BC2 coefficient, a leave-one-covariate-out sensitivity analysis was performed by refitting the model after separately omitting diabetes mellitus, baseline BM/week, or sex. LEfSe and PICRUSt2 analyses were retained as exploratory, hypothesis-generating analyses and were not used to define the primary response-associated taxa.

## 3 Result

### 3.1 Participant flow and baseline characteristics

A total of 29 participants were initially enrolled. Two participants did not complete the prescribed acupressure intervention, and one participant changed the laxative regimen during the study period; these three participants were excluded from the present responder analysis. The remaining 26 participants were included in the final analysis, of whom 9 (34.6%) met the prespecified response criterion and were classified as responders (R group), whereas 17 (65.4%) were classified as non-responders (NR group).

The R group included a higher proportion of women than the NR group (66.7% vs. 23.5%, P = 0.046). Baseline bowel movement frequency was numerically higher in responders [1.50 (1.00, 1.50) vs. 1.00 (0.50, 1.50) BM/week, P = 0.055], and diabetes mellitus was less frequent in the R group than in the NR group (44.4% vs. 82.4%, P = 0.078), although the latter differences did not reach statistical significance. No statistically significant between-group differences were observed in age, dialysis vintage, dialysis modality, BMI, Kt/V, baseline PAC-SYM scores, serum calcium or phosphorus, hypertension, or laxative use (all P > 0.05; **Table 1**).

**Table 1:** Baseline characteristics of responders and non-responders.

Variable	Overall (n=26)	R (n=9)	NR (n=17)	P value
Age, years	67.0 (57.25, 72.50)	69.0 (53.0, 71.0)	66.0 (62.0, 73.0)	0.746
Sex, male/female	16/10	3/6	13/4	0.046
Dialysis vintage, years	3.0 (1.25, 6.75)	2.0 (2.0, 4.0)	4.0 (1.0, 7.0)	0.624
Dialysis frequency, sessions/week	3	3	3	—
Conventional HD, n (%)	4 (15.4)	2 (22.2)	2 (11.8)	0.591
BMI, kg/m²	22.73 (20.81, 24.57)	22.04 (20.73, 24.53)	23.42 (21.36, 24.97)	0.706
Kt/V	1.49 (1.34, 1.67)	1.62 (1.39, 1.72)	1.40 (1.20, 1.60)	0.131
Diabetic kidney disease, n (%)	12 (46.2)	2 (22.2)	10 (58.8)	0.110
Hypertension, n (%)	24 (92.3)	9 (100)	15 (88.2)	0.529
Diabetes mellitus, n (%)	18 (69.2)	4 (44.4)	14 (82.4)	0.078
Laxative use, n (%)	8 (30.8)	3 (33.3)	5 (29.4)	1.000
Baseline BM, BM/week	1.25 (1.00, 1.50)	1.50 (1.00, 1.50)	1.00 (0.50, 1.50)	0.055
PAC-SYM total score	1.96 (1.83, 2.23)	1.92 (1.83, 2.08)	2.08 (1.92, 2.50)	0.116
PAC-SYM stool-symptom score	2.40 (2.20, 2.60)	2.20 (2.20, 2.40)	2.40 (2.20, 2.80)	0.394
Serum calcium, mmol/L	2.17 (2.09, 2.29)	2.15 (2.08, 2.29)	2.18 (2.11, 2.28)	0.978
Serum phosphorus, mmol/L	1.45 (1.20, 1.85)	1.57 (1.29, 1.63)	1.44 (1.20, 1.88)	0.914

Data are presented as median (Q1, Q3) or n (%). Continuous variables were compared using the Mann–Whitney U test and categorical variables using Fisher’s exact test. BM, bowel movements; HD, hemodialysis; PAC-SYM, Patient Assessment of Constipation Symptoms.

### 3.2 Short-term clinical response

Across the 26 participants, weekly bowel movement frequency increased from 1.25 (1.00, 1.50) BM/week before the intervention to 2.00 (1.00, 3.00) BM/week during the second intervention week (P = 0.0003). The PAC-SYM total score decreased from 1.96 (1.83, 2.23) to 1.83 (0.92, 2.29) (P = 0.0011), and the stool-symptom subscale score decreased from 2.40 (2.20, 2.60) to 2.00 (1.20, 2.40) (P = 0.0013). Nine of 26 participants (34.6%) met the prespecified short-term response criterion of an increase of at least 1 BM/week (**Table 2**).

**Table 2:** Changes in constipation-related clinical outcomes.

Outcome	Pre-intervention	Post-intervention	P value
BM, BM/week	1.25 (1.00, 1.50)	2.00 (1.00, 3.00)	0.0003
PAC-SYM total score	1.96 (1.83, 2.23)	1.83 (0.92, 2.29)	0.0011
PAC-SYM stool-symptom score	2.40 (2.20, 2.60)	2.00 (1.20, 2.40)	0.0013

Data are presented as median (Q1, Q3). Pre- and post-intervention comparisons were performed using the Wilcoxon signed-rank test.

### 3.3 Microbial diversity analysis

#### 3.3.1 α-diversity analysis

Rarefaction curves approached a plateau in both groups, suggesting that sequencing depth was sufficient to capture the major microbial diversity present in the samples. Alpha-diversity was further assessed using the Shannon, Simpson, and Chao1 indices. No statistically significant differences in these indices were observed between responders and non-responders (all P > 0.05).

#### 3.3.2 β-diversity analysis

Beta-diversity analysis showed substantial overlap between responders and non-responders. Principal coordinates analysis (PCoA) based on Bray-Curtis distances showed no clear separation between the two groups. Similarly, non-metric multidimensional scaling (NMDS) based on unweighted UniFrac distances showed considerable overlap in sample distribution. Consistent with these ordination results, ANOSIM showed no significant difference in overall microbial community structure between the R and NR groups (R = −0.044, P = 0.6751) (**Figure 1**).

**Figure 1:**
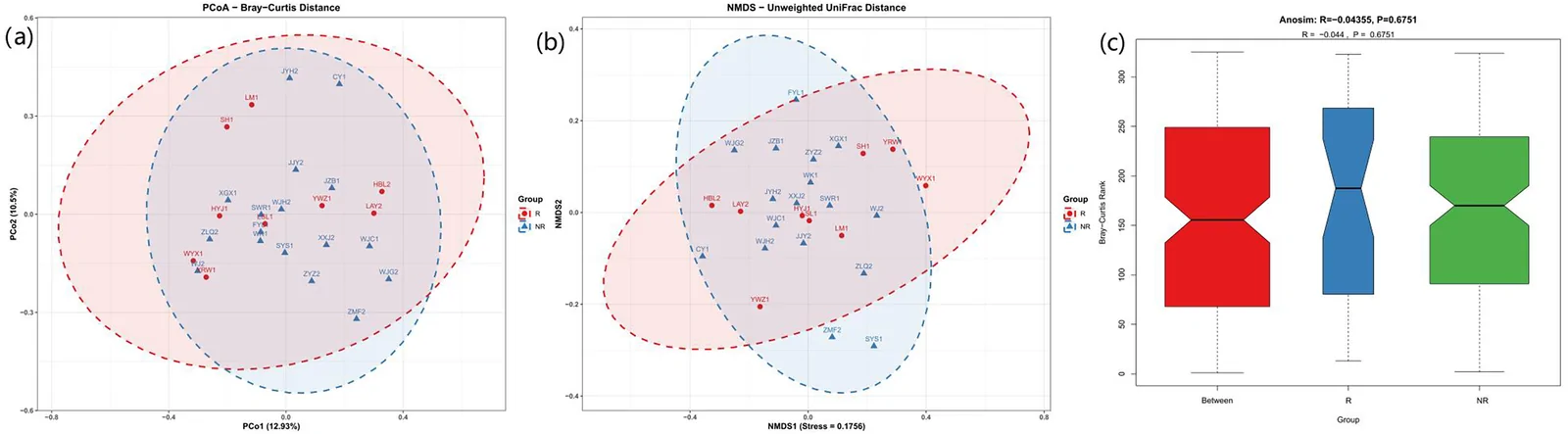
Baseline gut microbiota β-diversity in responders and non-responders. β-diversity of baseline gut microbiota in responders (R, n = 9) and non-responders (NR, n = 17). **(a)** PCoA based on Bray–Curtis dissimilarity; **(b)** NMDS based on unweighted UniFrac distance; **(c)** ANOSIM based on Bray–Curtis dissimilarity (R = −0.044, P = 0.6751).

### 3.4 Taxonomic composition and differential abundance analysis

#### 3.4.1 Overall taxonomic composition

At the genus level, the baseline gut microbiota showed substantial interindividual variation in both responders and non-responders, while the overall composition of the dominant genera was broadly similar between the two groups (**Figure 2**). The most abundant genera across the study population included *Blautia, Streptococcus, Bifidobacterium*, [Eubacterium]_hallii_group, Ligilactobacillus, *Escherichia–Shigella, Limosilactobacillus, Anaerostipes, Collinsella*, and *Faecalibacterium*. Among these, Blautia and Streptococcus were predominant in both groups. Descriptively, responders showed relatively higher abundances of Anaerostipes and Faecalibacterium, whereas *Bifidobacterium, Ligilactobacillus, Escherichia–Shigella*, and *Limosilactobacillus* tended to be more abundant in non-responders. However, considerable variation was observed among individual participants, and these descriptive differences were not interpreted as evidence of statistically significant group effects. Differential abundance was therefore evaluated separately using unadjusted and multivariable analyses.

**Figure 2:**
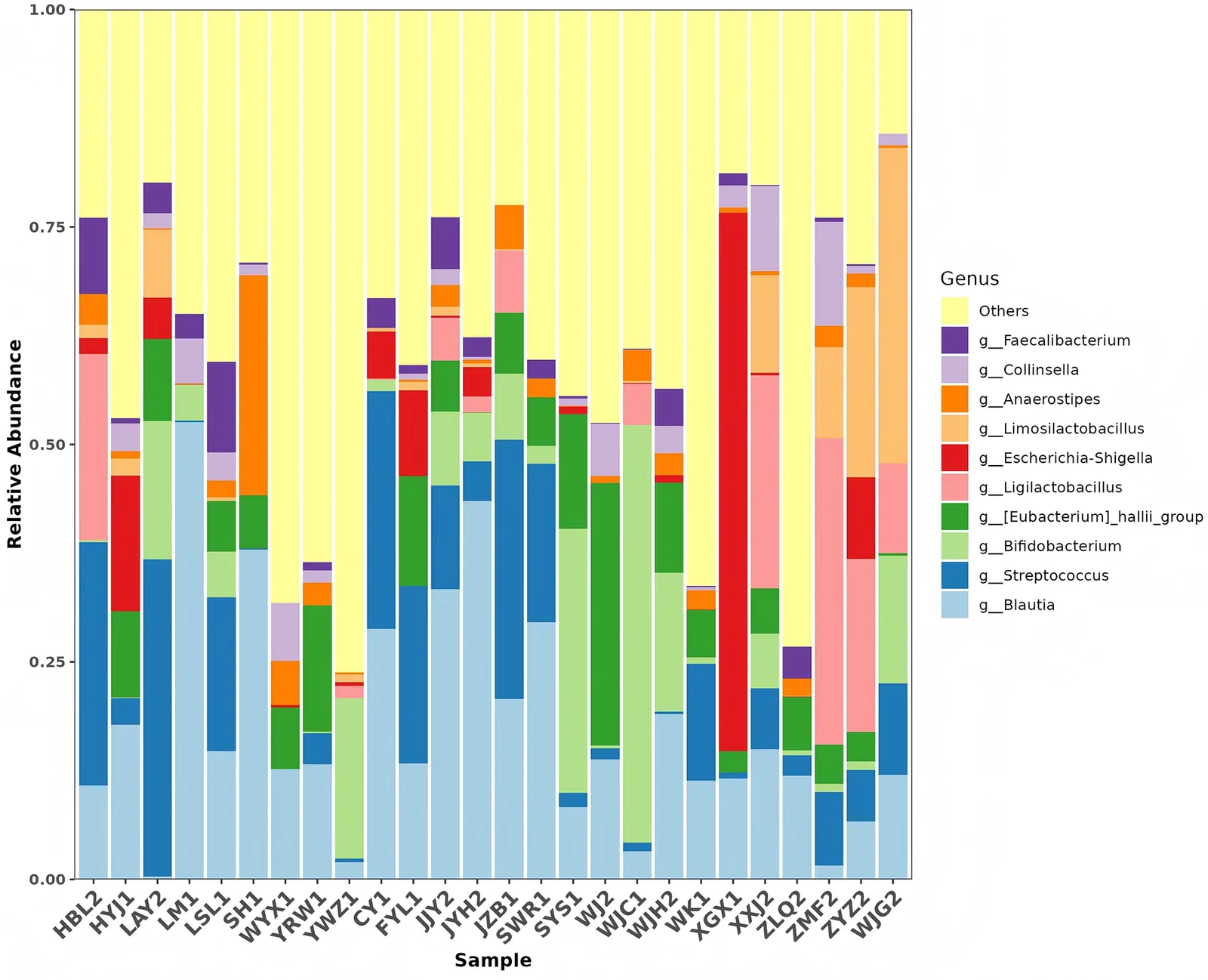
Genus-level composition of the baseline gut microbiota. Relative abundances of the dominant genera in individual baseline fecal samples. Each bar represents one participant, and genera not individually displayed are grouped as “Others”.

#### 3.4.2 Unadjusted taxonomic comparisons

At the phylum level, the overall taxonomic composition of the baseline gut microbiota was broadly similar between responders and non-responders. Firmicutes, Actinobacteriota, Proteobacteria, and Bacteroidota were the predominant phyla in both groups, whereas several other phyla were present at relatively low abundances. No statistically significant between-group differences were observed in the relative abundances of the major phyla (all P > 0.05).

At the genus level, the overall abundance profiles were likewise broadly comparable between the two groups. In the unadjusted Wilcoxon rank-sum analysis, *Lactonifactor* showed a nominally higher relative abundance in responders than in non-responders (P = 0.015). However, this association did not remain significant after Benjamini–Hochberg correction for multiple testing (FDR-adjusted P = 0.86). No other genus showed a nominal P < 0.05 in the Wilcoxon analysis (**Figure 3**). Accordingly, the *Lactonifactor* finding was considered an exploratory unadjusted signal rather than a statistically robust response-associated taxon. Genus-level differential abundance was subsequently evaluated using multivariable ANCOM-BC2 analysis with adjustment for potential clinical confounders and false-discovery-rate correction.

**Figure 3:**
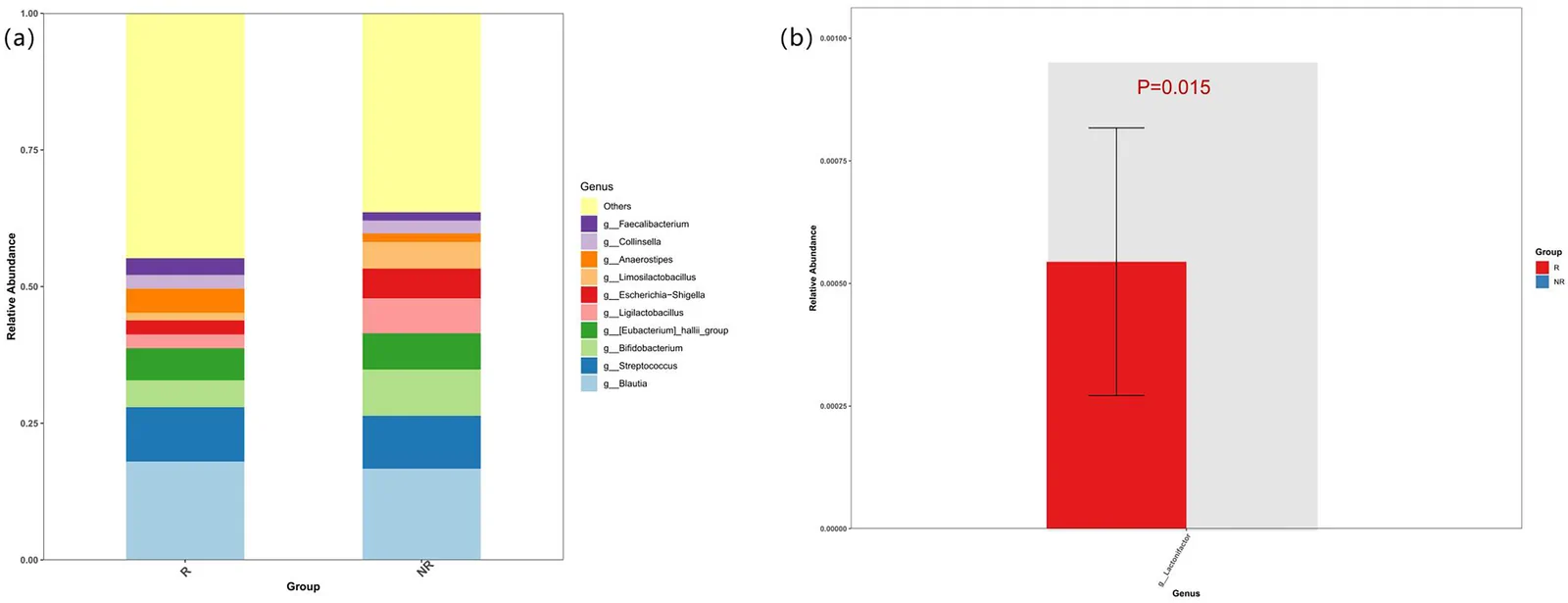
Genus-level taxonomic composition and unadjusted differential abundance between responders and non-responders. **(a)** Genus-level relative abundance profiles in responders (R) and non-responders (NR). **(b)** Unadjusted relative abundance of *Lactonifactor*, which was nominally higher in responders (P = 0.015) but was not significant after Benjamini–Hochberg correction (FDR-adjusted P = 0.86).

#### 3.4.3 Multivariable differential abundance analysis

Genus-level differential abundance was further evaluated using ANCOM-BC2 to account for potential clinical confounding, the compositional nature of microbiome data, and multiple testing. After filtering for prevalence (≥20%) and mean relative abundance (≥0.01%), 81 genera were retained for analysis. The multivariable model included responder status as the primary variable and adjusted for baseline BM/week, sex, and diabetes mellitus.

After filtering for prevalence (≥20%) and mean relative abundance (≥0.01%), 81 genera were retained. After adjustment for baseline BM/week, sex, and diabetes mellitus and Benjamini–Hochberg correction for multiple testing, *Veillonella* and *Lactobacillus* met the FDR <0.05 threshold in the primary ANCOM-BC2 model. *Veillonella* was positively associated with responder status (β = 5.319, SE = 0.496, P = 0.00043, FDR = 0.0347), and *Lactobacillus* also showed a positive adjusted association (β = 3.621, SE = 0.542, P = 0.00113, FDR = 0.0459). Gordonibacter showed a positive association but did not meet the prespecified FDR threshold (β = 2.921, P = 0.00189, FDR = 0.0511). No other tested genus met FDR <0.05 (**Table 3**). The unadjusted baseline relative abundances of Veillonella and Lactobacillus in the R and NR groups are shown descriptively in **Figure 4**.

**Table 3:** Multivariable ANCOM-BC2 analysis of baseline genus-level differential abundance associated with responder status.

Genus	β	SE	P value	FDR	Pseudocount sensitivity
*Veillonella*	5.319	0.496	0.00043	0.0347	Not robust
*Lactobacillus*	3.621	0.542	0.00113	0.0459	Not robust
*Gordonibacter*	2.921	—	0.00189	0.0511	—

β coefficients represent the adjusted association between genus abundance and responder status, with the non-responder group as the reference. The model was adjusted for baseline bowel movement frequency, sex, and diabetes mellitus. Benjamini-Hochberg correction was applied for multiple testing, with FDR < 0.05 considered statistically significant. Pseudocount sensitivity analysis was used to assess robustness to zero handling. β, regression coefficient; SE, standard error; FDR, false discovery rate; BM, bowel movement. Only genera with FDR < 0.10 are shown for concise presentation.

**Figure 4:**
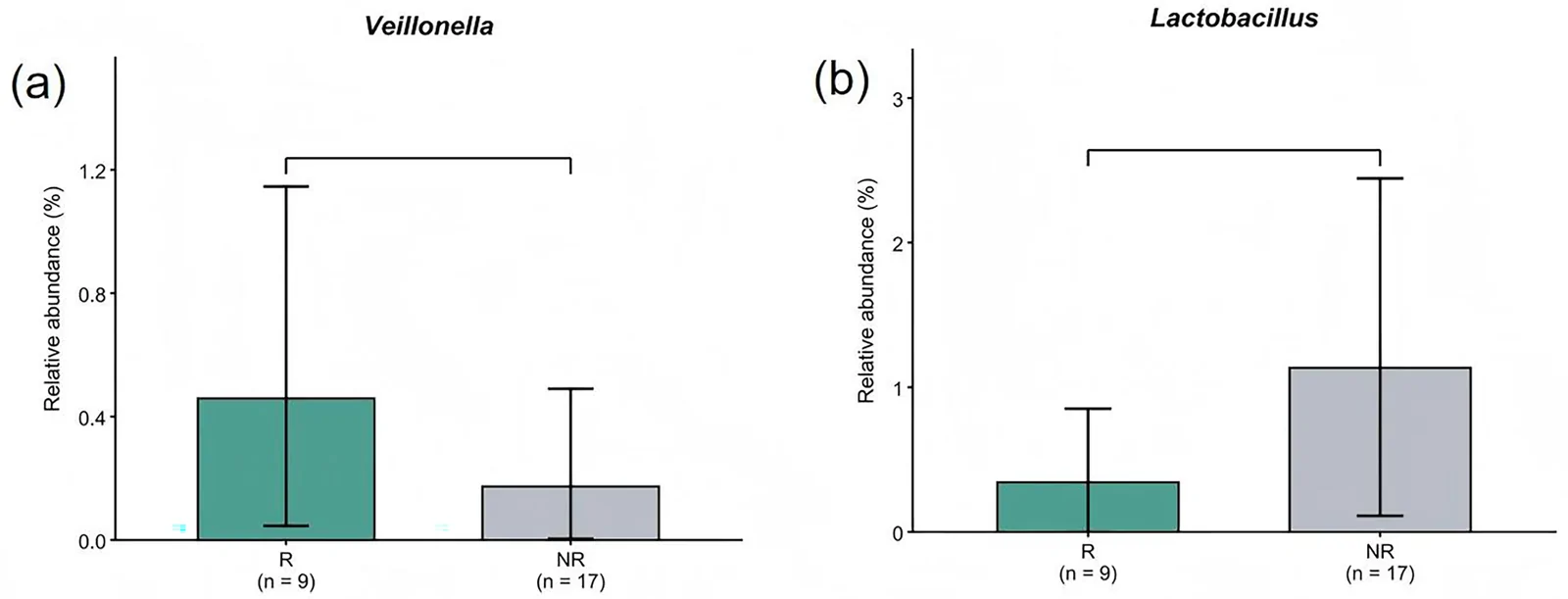
Baseline relative abundances of *Veillonella* and *Lactobacillus* in responders and non-responders. Descriptive baseline relative abundances of **(a)** *Veillonella* and **(b)** *Lactobacillus* in responders (R, n = 9) and non-responders (NR, n = 17). Error bars represent 95% confidence intervals. These plots show unadjusted descriptive relative abundances; adjusted associations with responder status were evaluated separately using ANCOM-BC2.

Neither the *Veillonella* nor the *Lactobacillus* association remained robust in the ANCOM-BC2 pseudocount sensitivity analysis. Moreover, the direction of the adjusted *Lactobacillus* association differed from that observed in the unadjusted descriptive relative abundance data. Leave-one-covariate-out analyses further showed that the magnitude, direction, and statistical significance of the *Lactobacillus* association varied across reduced models, indicating sensitivity to covariate specification. Accordingly, these findings should be interpreted as exploratory response-associated signals rather than robust microbial biomarkers.

### 3.5 Exploratory LEfSe and functional prediction analyses

Exploratory LEfSe analysis identified several taxa with differential representation between responders and non-responders, including Erysipelotrichales, *Citrobacter*, *Raoultella*, *Eubacterium ramulus*, *Lactonifactor*, and *Morganella*, among others. Because LEfSe did not provide the same level of covariate adjustment and multiple-testing control as the primary ANCOM-BC2 analysis, these findings were considered hypothesis-generating and were not interpreted as independently validated response-associated taxa.

PICRUSt2-based functional prediction showed broadly similar predicted functional profiles between the R and NR groups. Although several pathways showed numerical differences, none reached statistical significance (all P>0.05). These predicted functional results were therefore considered exploratory and require confirmation using direct functional approaches such as metagenomics or metabolomics.

## 4 Discussion

Maintenance hemodialysis is a long-term kidney replacement treatment for patients with end-stage kidney disease, requiring repeated extracorporeal removal of retained solutes and excess fluid. Constipation in patients receiving MHD refers to persistent difficulty with defecation in this specific clinical setting and may manifest as infrequent bowel movements, hard stools, straining, incomplete evacuation, and prolonged intestinal transit. Objective transit studies have shown that total colonic transit time is significantly prolonged in long-term hemodialysis patients compared with healthy controls, suggesting that altered intestinal motility is an important component of constipation in this population (Wu et al., 2004). This is particularly relevant to gut microbiota research because intestinal transit and stool characteristics are closely related to fecal microbial richness, community composition, and bacterial growth dynamics (Vandeputte et al., 2016). In our previous study, MHD patients with constipation showed lower microbial diversity and distinct microbial profiles compared with MHD patients without constipation and healthy controls (Zhang et al., 2024a). These findings suggest that gut microbiota constitute part of the biological background of constipation in MHD patients.

In the present study, bowel movement frequency increased and both PAC-SYM total and stool-symptom subscale scores decreased during the 14-day intervention with acupressure combined with thermal stimulation. However, only 9 of 26 participants (34.6%) met the predefined bowel-movement-based response criterion. This relatively modest proportion is more appropriately interpreted as evidence of marked short-term response heterogeneity than as a simple estimate of overall treatment efficacy, particularly because the response criterion was based on an increase of ≥1 BM/week and the observation period was only 14 days. Previous evidence also suggests that acupressure can improve constipation in the hemodialysis population. In a randomized double-blind controlled trial involving 70 hemodialysis patients, Abbasi et al. reported significant improvement in defecation frequency and stool characteristics after four weeks of acupressure compared with stimulation at control points (Abbasi et al., 2019). Differences in treatment duration, acupoints, intervention procedures, and outcome definitions preclude direct comparison of response rates; nevertheless, these findings support the clinical relevance of acupressure for bowel dysfunction in this population. The variability observed in our study therefore provided an opportunity to investigate why patients receiving the same intervention may experience different degrees of short-term improvement.

An important finding from recent functional-constipation research is that such treatment heterogeneity may be related to the pretreatment intestinal microbiota. Lai et al. conducted a double-blind randomized placebo-controlled trial involving 250 adults with functional constipation who received different dietary fiber or probiotic interventions. Although the overall microbial diversity and community structure were broadly comparable among intervention groups, intervention-specific baseline microbial genera were able to distinguish subsequent responders from non-responders, indicating that the pretreatment microbial environment may influence individual responsiveness (Lai et al., 2023). Similarly, Zhang et al. investigated 104 patients with functional constipation treated with a defined multi-strain probiotic preparation and reported an overall response rate of 54.8%. In the subgroup undergoing metagenomic sequencing, baseline PCoA did not demonstrate clear separation between responders and non-responders, although differences in selected microbial features and ecological networks were observed (Zhang et al., 2024b).

Our findings are consistent with this pattern. No statistically significant differences were detected between responders and non-responders in the evaluated α-diversity indices, and β-diversity analyses likewise showed substantial overlap between groups, with ANOSIM showing no evidence of overall community separation (R = −0.044, P = 0.6751). Thus, the present data do not support the existence of a globally distinct baseline “responder microbiome.” This differs from our previous comparison between MHD patients with and without constipation, in which broader differences in microbial diversity and taxonomic composition were observed (Zhang et al., 2024a). Considered together, these observations suggest that the microbial differences associated with the presence of constipation may be broader than those associated with subsequent response among patients who already have constipation. In the latter setting, treatment heterogeneity may be reflected by more subtle taxon-level characteristics rather than by global differences in community diversity.

This distinction became clearer after multiple-testing correction and multivariable analysis. In the unadjusted Wilcoxon analysis, *Lactonifactor* was nominally more abundant in responders than in non-responders (P = 0.015); however, the association disappeared after Benjamini–Hochberg correction (FDR-adjusted P = 0.86). Therefore, the unadjusted Lactonifactor finding cannot be considered statistically robust. When genus-level differential abundance was subsequently evaluated using ANCOM-BC2 with adjustment for baseline BM/week, sex, and diabetes mellitus, *Veillonella* (β = 5.319, FDR = 0.0347) and *Lactobacillus* (β = 3.621, FDR = 0.0459) met the FDR <0.05 threshold, whereas *Gordonibacter* narrowly missed statistical significance. Importantly, neither the *Veillonella* nor the *Lactobacillus* association remained robust in the pseudocount sensitivity analysis. Moreover, the direction of the adjusted *Lactobacillus* association differed from that observed in the unadjusted descriptive relative abundances. These results indicate that the observed genus-level associations are sensitive to analytical assumptions and should therefore be interpreted as exploratory response-associated signals rather than established microbial biomarkers or independent predictors.

Among the genera identified in the adjusted analysis, *Veillonella* is of particular interest because of its metabolic niche within the intestinal microbial community and its previous association with constipation-related changes in the colonic environment. *Veillonella* species are anaerobic lactate-utilizing bacteria that can convert lactate generated by other intestinal microorganisms into short-chain fatty acids, particularly propionate and acetate, thereby participating in microbial cross-feeding networks rather than acting primarily as carbohydrate degraders themselves (Ng and Hamilton, 1971; Zhang and Huang, 2023). Experimental work has further shown that this lactate-utilizing and short-chain-fatty-acid-producing capacity is environmentally dependent, with substantial variation according to bacterial growth phase and nutrient availability, suggesting that the abundance of *Veillonella* may partly reflect the surrounding microbial metabolic context rather than a fixed functional effect (Zhang and Huang, 2023).

A potentially relevant clinical observation was reported by Jalanka et al., who studied patients with constipation receiving psyllium. Psyllium supplementation increased fecal water and was accompanied by changes in gastrointestinal transit, microbial composition, and fecal short-chain fatty acids; *Veillonella* was among the genera that increased after treatment, together with *Lachnospira, Faecalibacterium*, *Phascolarctobacterium*, and *Sutterella* (Jalanka et al., 2019). Notably, that study did not establish *Veillonella* itself as the organism responsible for improved bowel function, and the strongest reported associations with increased fecal water involved several butyrate-producing genera rather than *Veillonella*. Therefore, the finding is more appropriately interpreted as evidence that *Veillonella* can change as part of a broader microbial response accompanying alterations in intestinal transit and luminal conditions.

In the present study, the positive adjusted association between baseline *Veillonella* abundance and responder status may therefore indicate that responders entered the intervention with a somewhat different microbial metabolic background, potentially involving lactate utilization and cross-feeding relationships. However, this interpretation remains speculative. We did not measure fecal lactate, propionate, acetate, intestinal transit time, or other microbial metabolites, and the *Veillonella* association did not remain robust in the ANCOM-BC2 pseudocount sensitivity analysis. Moreover, 16S rRNA gene sequencing provides genus-level information and cannot determine which *Veillonella* species or metabolic pathways were functionally active. Accordingly, the present finding should be regarded as an exploratory association that provides a biologically plausible direction for future study, rather than evidence that *Veillonella* directly enhances the response to acupressure combined with thermal stimulation.

The association involving *Lactobacillus* should be interpreted with particular caution. *Lactobacillus* is a taxonomically and functionally heterogeneous genus, and biological effects demonstrated for individual species or strains cannot be extrapolated directly to the genus as a whole. This is well illustrated by previous intervention studies in functional constipation. In a systematic review and meta-analysis of randomized controlled trials, Dimidi et al. reported that probiotics overall improved intestinal transit time and stool frequency, but the magnitude of benefit varied considerably according to the microorganism administered; for example, Bifidobacterium animalis subsp. lactis improved stool frequency, whereas *Lactobacillus* casei Shirota did not show a significant effect (Dimidi et al., 2014). These findings emphasize that the clinical relevance of *lactobacilli* is likely to be species- and strain-dependent rather than a uniform property of the genus. This distinction is particularly important in the present study because 16S rRNA gene sequencing provides limited resolution below the genus level (Johnson et al., 2019), and the descriptive relative abundance of *Lactobacillus* did not show the same direction as the adjusted ANCOM-BC2 estimate. Moreover, the association did not remain robust in the pseudocount sensitivity analysis. Therefore, our finding should not be interpreted as indicating that a higher abundance of *Lactobacillus* is inherently beneficial or that this genus directly enhances treatment response. Instead, it may reflect a broader baseline microbial context associated with response heterogeneity, which requires confirmation at the species or strain level and functional validation in larger cohorts.

Given the limited robustness of the taxon-level findings, the LEfSe results should be regarded as complementary and hypothesis-generating (Segata et al., 2011). LEfSe identified several taxa enriched in responders, including Erysipelotrichales, *Citrobacter, Raoultella, Eubacterium ramulus, Morganella*, and *Lactonifactor*. Some of these taxa have previously been linked to microbial metabolic activities that may influence the intestinal environment. For example, *E. ramulus* is capable of degrading dietary flavonoids into phenolic metabolites, whereas *Lactonifactor longoviformis* participates in the microbial conversion of dietary lignans to enterolignans (Simmering et al., 2002; Bess et al., 2020). Experimental studies have also shown that *Morganella morganii* can generate bioactive microbial metabolites capable of influencing intestinal physiology (Chen et al., 2019). However, these findings were derived from species-level experimental studies in settings different from the present cohort and cannot be directly extrapolated to the genus-level signals identified by 16S rRNA gene sequencing. Moreover, the coexistence of taxa with markedly different ecological characteristics argues against interpreting the responder-enriched LEfSe profile as a uniformly beneficial microbial pattern. These taxa should therefore be considered leads for future functional investigation rather than mechanistic explanations of treatment response.

PICRUSt2 did not identify statistically significant differences in predicted functional pathways between responders and non-responders, although modest trends were observed in several metabolic pathways. Because PICRUSt2 infers metagenomic functional potential from marker-gene data rather than directly measuring microbial genes, transcripts, proteins, or metabolites (Douglas et al., 2020), these pathway-level trends should not be assigned mechanistic significance. Accordingly, the present data do not demonstrate that differences in microbial metabolite production, inflammatory regulation, neurotransmitter signaling, or intestinal barrier function account for the observed heterogeneity in clinical response. Direct validation using shotgun metagenomics, metabolomics, or other functional approaches will be required.

Several limitations should be acknowledged. First, this was a small, single-center exploratory study involving only 26 participants, including nine responders, which limited statistical power and the stability of high-dimensional microbiome analyses. Although covariate-adjusted ANCOM-BC2 and multiple-testing correction were applied, the significant genus-level associations did not remain robust in sensitivity analyses and should therefore be interpreted as exploratory response-associated signals rather than established biomarkers. Second, the responder/non-responder comparison was outcome-defined, and the ≥1 BM/week threshold was used as an operational criterion for short-term response stratification; therefore, causal or predictive conclusions cannot be drawn. Third, despite adjustment for several clinically relevant factors and stable laxative regimens, residual confounding remains possible because the small sample size restricted the number of covariates that could be included. Fourth, only baseline microbiota were analyzed, preventing assessment of intervention-induced microbial changes or mediation effects. Finally, the 14-day intervention period was relatively short, the intervention was largely self-administered, and 16S rRNA gene sequencing with PICRUSt2 provided limited taxonomic and functional resolution. Larger prospective studies with longitudinal sampling, longer follow-up, and integrated metagenomic and metabolomic analyses are needed to validate these findings.

## 5 Conclusions

This exploratory study showed that 9 of 26 MHD patients with functional constipation met the predefined short-term response criterion after acupressure combined with thermal stimulation. Baseline α-diversity and overall microbial community structure did not differ significantly between responders and non-responders, suggesting that short-term response was not characterized by a globally distinct baseline microbiome. At the genus level, the unadjusted *Lactonifactor* association did not remain significant after multiple-testing correction, whereas *Veillonella* and *Lactobacillus* met the FDR threshold in the adjusted ANCOM-BC2 analysis but were not robust in pseudocount sensitivity testing. Therefore, these taxa should be regarded as exploratory response-associated signals rather than established microbial biomarkers or independent predictors. Larger prospective studies with longitudinal microbiota sampling and direct functional analyses are needed to determine whether baseline microbial characteristics provide clinically meaningful information on treatment-response heterogeneity.

## Data Availability

The data presented in the study are deposited in the National Center for Biotechnology Information (NCBI) BioProject repository, accession number PRJNA1417464.
